# PTBP1‐Mediated Alternative Splicing of DNAJB6 Promotes Everolimus Resistance in Clear Cell Renal Cell Carcinoma via EIF4B/PKIB/AKT/mTOR Positive Feedback Loop

**DOI:** 10.1002/advs.77782

**Published:** 2026-09-27

**Authors:** Xiu‐wu Pan, Hong‐feng Zheng, Mu‐chen Li, Jia‐lin Zhou, Hang‐biao Zhang, Dong‐hao Lyu, Yi‐fan Tang, Tian‐yue Yang, Yi‐fan Liu, Zi‐chang Liu, Wen‐jie Ma, Yuan‐bo Zong, Jian‐gui Liu, Zi‐xuan Gong, Hao Zhang, Ke‐qin Dong, Wang Zhou, Jian‐qin Ye, Xin‐gang Cui

**Affiliations:** ^1^ Department of Urology Xinhua Hospital School of Medicine Shanghai Jiao Tong University Shanghai China; ^2^ Department of Neurosurgery Naval Medical Center of PLA Second Military Medical University Shanghai China

**Keywords:** alternative splicing, clear cell renal cell carcinoma, DNAJB6b, drug resistance, everolimus, PTBP1

## Abstract

**Background**: Everolimus (EVE) is a key treatment for advanced clear cell renal cell carcinoma (ccRCC), mainly targeting AKT/mTOR signaling. However, developed resistance remains a major challenge for EVE treatment. Aberrant alternative splicing, particularly regulated by PTBP1, may represent a critical mechanism enabling the activation of these resistance‐associated pathways.

**Methods**: Single‐cell transcriptome sequencing is performed to reveal the association between PTBP1 expression, EVE resistance and AKT/mTOR pathway activity. Patient‐derived specimens, animal models, and in vitro assays are used to evaluate the role of PTBP1 in EVE resistance. Full‐length transcriptome sequencing and crosslinking immunoprecipitation (CLIP) sequencing are employed to identify PTBP1‐regulated splicing targets. Co‐immunoprecipitation, proteomic analyses, and molecular docking further elucidate the mechanism by which DNAJB6b re‐activated AKT/mTOR signaling.

**Results**: PTBP1 is upregulated in EVE‐resistant tumors and its expression positively correlates with AKT/mTOR signaling activation. PTBP1 promotes the expression of the DNAJB6b isoform through alternative splicing. EIF4B is identified as a direct binding partner of DNAJB6b, forming a complex that enhances oncogenic translation and activates a PKIB‐dependent AKT/mTOR signaling cascade.

**Conclusion**: This study demonstrates that PTBP1 drives EVE resistance in ccRCC by upregulating DNAJB6b, which recruits EIF4B to initiate a PKIB/AKT/mTOR positive feedback loop, sustaining oncogenic translation and therapeutic evasion.

## Graphical abstract

PTBP1‐mediated alternative splicing drives a pathogenic switch to the DNAJB6b isoform in everolimus‐resistant clear cell renal cell carcinoma. This splicing event programs a positive feedback loop including EIF4B/PKIB/AKT/mTOR, promoting aggressive therapy resistance.

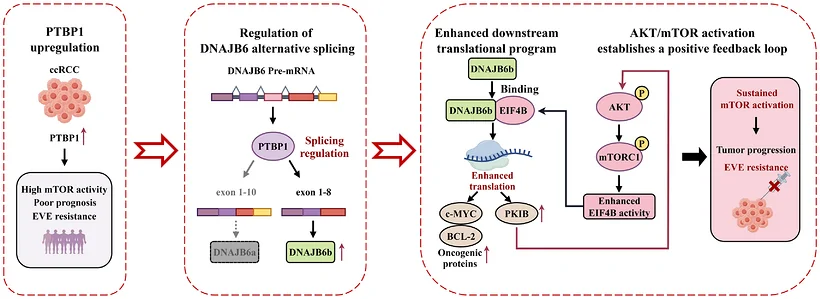

## 1 Introduction

Approximately one‐third of clear cell renal cell carcinoma (ccRCC) patients present with advanced stage at initial diagnosis, often requiring systemic therapeutic interventions [1]. Everolimus (EVE), an inhibitor of mammalian target of Rapamycin (mTOR), is widely utilized in the second‐line treatment for ccRCCs [2], but its efficacy is limited by frequent acquired resistance, leaving patients with few effective options [3, 4, 5]. Consequently, elucidating the mechanisms underlying this resistance is therefore critical for developing improved therapeutic strategies.

Resistance to mTOR inhibitors involves complex mechanisms, given mTOR's central role in the PI3K/AKT/mTOR network [6]. Emerging evidence implicates crosstalk and feedback regulation between mTOR and AKT as key drivers of resistance [7, 8, 9]. Nevertheless, the precise regulatory relationships within this axis remain unclear, and their clarification may reveal novel approaches to overcome EVE resistance in ccRCC.

Alternative splicing (AS) is a post‐transcriptional regulatory mechanism [10, 11]. In cancer, aberrant AS can produce oncogenic isoforms or disrupt tumor suppressors, often driven by mutations in splicing elements or alterations in splicing factors [12, 13, 14, 15]. Recent studies have shown that splicing factors can promote tumor progression by modulating AKT/mTOR signaling activity [16, 17, 18, 19], potentially contributing to therapy resistance.

PTBP1, a member of polypyrimidine tract binding protein family, plays an essential role in pre‐mRNA processing and maturation. PTBP1 was found to promote tumor progression and metastasis in colorectal cancer and hepatocellular carcinoma by extensive AS [20, 21]. Besides, previous studies reported that PTBP1 was involved in PI3K/AKT/mTOR signaling [22, 23, 24]. However, specific mechanism by which PTBP1 regulated AKT/mTOR pathway and influenced EVE resistance in ccRCC still remained unknown.

In this study, we found PTBP1 expression was increased in EVE‐resistant and high‐mTOR activity ccRCCs. High expression of PTBP1 was correlated to poor prognosis in patients with ccRCC. Using PTBP1‐knockout cells coupled with full‐length transcriptome sequencing, and cross‐link immunoprecipitation sequencing (CLIP‐seq), we identified DNAJB6 pre‐mRNA as direct downstream targets of PTBP1. Mechanistically, we found that PTBP1 upregulates a specific isoform of DNAJB6 (isoform b), which interacts with EIF4B and formed a complex. On one hand, EIF4B functions as a downstream effector of mTOR to sustain oncogenic translation. On the other hand, this complex upregulates PKIB, which in turn maintains persistent activation of the AKT/mTOR pathway via a positive feedback loop, thereby facilitating the development of EVE resistance. This study thus uncovers a previously unrecognized PTBP1‐dependent splicing mechanism driving mTOR inhibitor resistance in ccRCC and elucidates a novel molecular mechanism underlying the feedback activation between mTOR and AKT.

## 2 Results

### 2.1 PTBP1 is Upregulated in EVE‐Resistant Tumor and Correlates With High mTOR Activity

**FIGURE 1:**
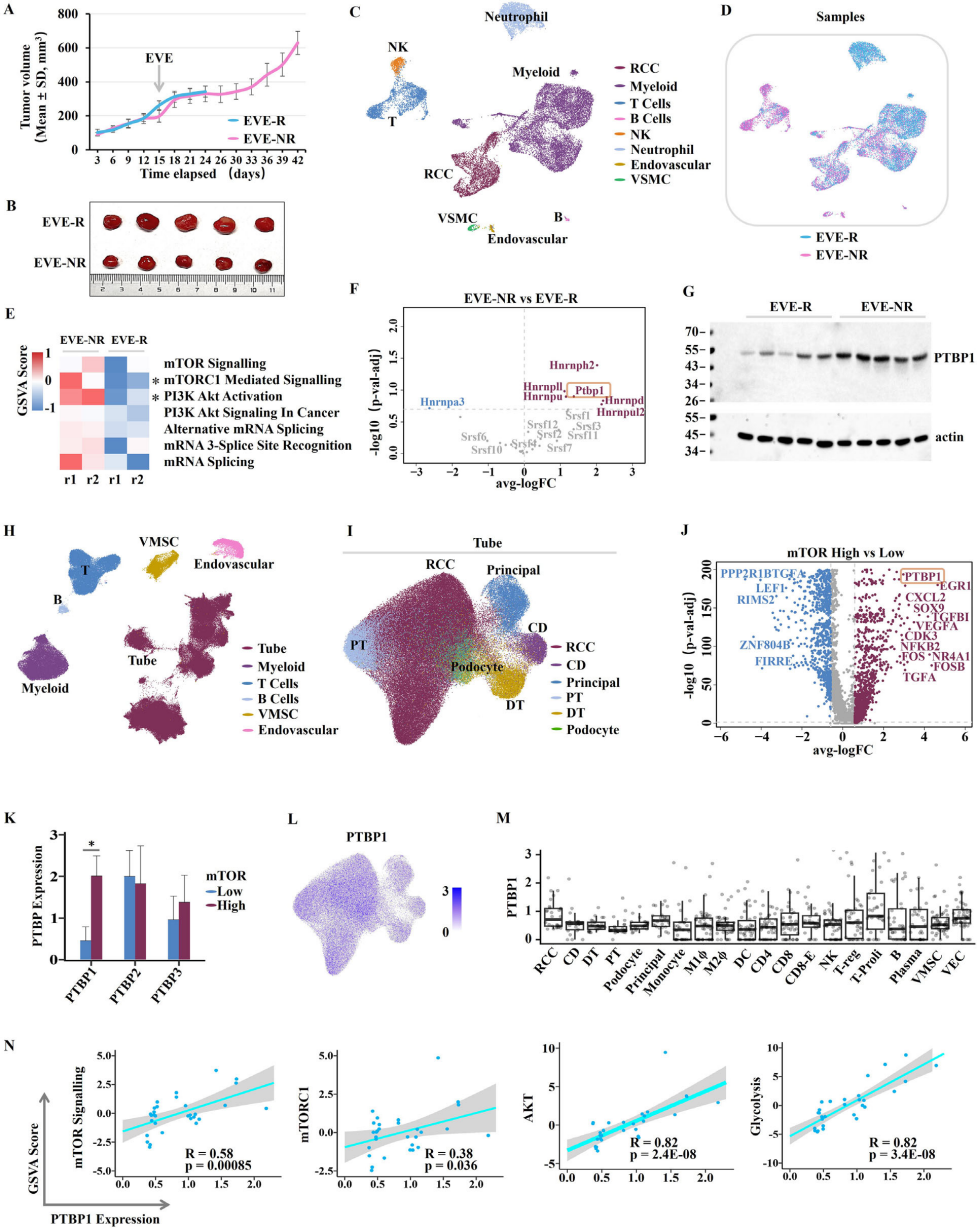
PTBP1 is upregulated in EVE‐resistant tumor and correlates with high mTOR activity. (A) Growth curve of Renca tumors from EVE responsive stage and non‐responsive stage. (B) Tumors harvested from responsive stage and non‐responsive stage. (C) The cell type clusters of all cells from Renca tumors. (D) The group clusters of all cells. (E) Heatmap of GSVA score of tumor cells from 4 samples. (F) Differential genes analysis of splicing factors between EVE‐responsive and ‐non‐responsive stage (right: upregulated in EVE‐NR group). (G) PTBP1 was upregulated in EVE‐non‐responsive group compared to responsive group. (H) The cell type clusters of all cells from human tissues. (I) The cell type clusters of epithelial cells. (J) Volcano plot of differentially expressed genes between mTOR‐high activity tumors and low activity tumors. (K) PTBP family members expression in two group (red: upregulated in mTOR‐High group). (L) Featureplot of PTBP1 in epithelial cell populations. (M) PTBP1 expression in all cell population. (N) PTBP1 was highly correlated with AKT/mTOR signaling. Statistical tests involved: *P < 0.05, **P < 0.01, ***P < 0.001, Student's t‐test; Data are expressed as mean ± SD, *n* = 3.

To explore the factor leading to resistance to EVE therapy, we constructed an animal model (Figure 1A). Renca tumor cells were injected subcutaneously to balb/c mice, and then the mice were treated with EVE. In the early stage, tumor grew at a low rate, which was considered as responsive stage (EVE‐R). However, after a period of treatment, the growth rate of the tumor accelerated significantly, which we attribute to the development of drug resistance (EVE‐NR, Figure 1B). We further performed single‐cell transcriptome on tumors from these two stages, respectively (Figure 1C,D and Figure S1A,B). We focused on the tumor cells and result of GSVA analysis showed that PI3K/AKT/mTOR related pathways were obviously activated in EVE‐NR group, due to developed resistance rendering them no longer inhibited by EVE (Figure 1E, Figure S1C). Notably, pathways associated with mRNA splicing were also enriched, though without statistical significance. Further differential gene expression analysis on splicing factors revealed that several members were significantly upregulated in the drug‐resistant group (Figure 1F). Among them, PTBP1 was the sole alternative splicing factor. Additionally, western blotting assay showed PTBP1 protein was increased in non‐responsive group (Figure 1G).

Subsequently, we extended our study in human‐derived tissues. Single‐nucleus RNA sequencing data of 30 cases of ccRCC tissues and 24 cases of normal kidney tissues were integrated and all cells were clustered into 6 main subsets (Figure 1H, Figure S1D,E). Further, tube compartment was divided into several types and we focused on tumor cells (Figure 1I and Figure S1F,G). Through pathway enrichment analysis, we divided all ccRCC cells into mTOR‐high activity group and mTOR‐low activity group. Consistently, we noticed PTBP1 was one of the most significantly upregulated genes in mTOR‐high group (Figure 1J). Previous studies have reported an association between PTBP1 and AKT/mTOR pathway activity in cancer [24, 25], but precise molecular mechanism remains unknown. Acting as a main member of core spliceosome, PTBP1 had two homologous members, PTBP2 and PTBP3. However, PTBP2 and PTBP3 did not show difference between two groups (Figure 1K). PTBP1 had a wide range of expression in all cell population (Figure 1L). Notably, a significant upregulation was found in ccRCC cells compared to normal tubular epithelium (Figure 1M). Additionally, higher expression of PTBP1 was correlated with higher GSVA score of mTOR signal pathway, mTORC1, AKT and glycolysis (Figure 1N).

### 2.2 PTBP1 Correlates With Clinical Prognosis and Modulates Response to EVE

**FIGURE 2:**
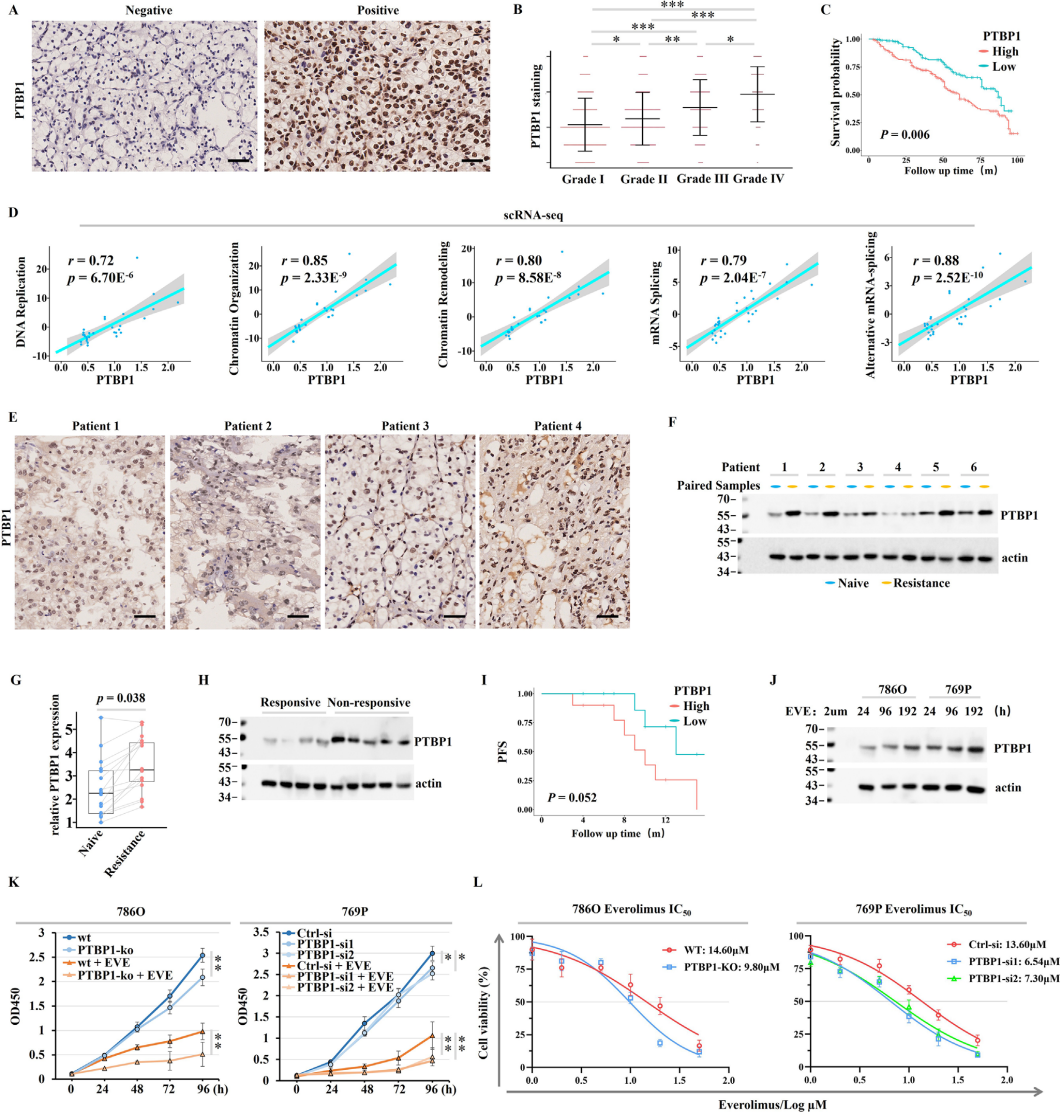
PTBP1 correlates with clinical prognosis and modulates response to EVE, scale bar = 50 µm. (A) Representative immunohistochemical images of PTBP1 in patients ccRCC tissues. (B) Association between PTBP1 staining score (from 0 to 6) and WHO/ISUP grade. (C) Kaplan‐Meier curves of overall survival probability in Xinhua Hospital cohort (*n* = 393, high group = 209, low group = 184). (D) Correlation analysis between PTBP1 expression and DNA replication, chromatin organization, chromatin remodeling, mRNA spicing and alternative splicing. (E) Representative immunohistochemical images of PTBP1 in ccRCC tissues from patients resistant to EVE, scale bar = 50 µm. PTBP1 was upregulated after resistance to EVE developed compared to before treatment, which was assessed by western blotting (F) and immunohistochemical staining (G). (H) PTBP1 expression was higher in treatment‐responsive patients than in non‐responders. (I) Kaplan‐Meier curves of progression‐free survival probability in EVE‐treatment cohort (*n* = 20, high group = 10, low group = 10). (J) Long‐term exposure to EVE increased the PTBP1 expression in ccRCC cells. (K) Inhibition of PTBP1 could weaken the proliferation rate of ccRCC cells, and could cooperate with EVE treatment. (L) inhibition of PTBP1 could increase the sensitivity to EVE treatment. Statistical tests involved: *P < 0.05, **P < 0.01, ***P < 0.001, Student's t‐test; Data are expressed as mean ± SD, *n* = 3.

We analyzed the PTBP1 expression in patient tissues and found it located in cell nucleus (Figure 2A). The expression level of PTBP1 was positively correlated with the overall nuclear grade of the tumor (Figure 2B), and its higher expression led to poor overall survival of ccRCC patients (Figure 2C). Through analyzing scRNA‐seq and TCGA‐KIRC database, PTBP1 was found positively correlated with several nucleus‐related functions in addition to splicing, which indicated its more complicated role (Figure 2D, Figure S2). Notably, PTBP1 exhibited high expression in tissue samples from EVE‐resistant patients (Figure 2E). By comparing paired samples before and after treatment, PTBP1 expression was found significantly higher after the development of drug resistance than before treatment (Figure 2F,G), underscoring the important role of its dynamic changes in the development of drug resistance. Furthermore, in EVE responsive group, PTBP1 expression was lower than non‐responsive group (Figure 2H). Patients with higher baseline PTBP1 expression showed worse progression‐free survival than those with lower expression (Figure 2I). These results indicate that PTBP1 may serve as a potential predictive biomarker.

Subsequently, we investigated PTBP1 in in vitro assay. We observed that PTBP1 expression was upregulated in cells following prolonged exposure to EVE (Figure 2J), which is consistent with our in vivo model. To thoroughly explore the effect of PTBP1 in ccRCC, we constructed PTBP1 knockout 786O cells (PTBP1‐KO) by CRISPR‐Cas9. Compared to wild type (WT) cells, PTBP1‐KO showed weaker ability of proliferation, migration and invasion in vitro (Figure S3A–C). Similar results were found in PTBP1 knockdown 769P cells. Furthermore, PTBP1‐KO and PTBP1‐knockdown cells were more sensitive to EVE treatment (Figure 2K,L). These findings further suggested that PTBP1 upregulation and developed EVE resistance are likely mutually reinforcing.

### 2.3 PTBP1 Regulates Alternative Splicing in ccRCC

**FIGURE 3:**
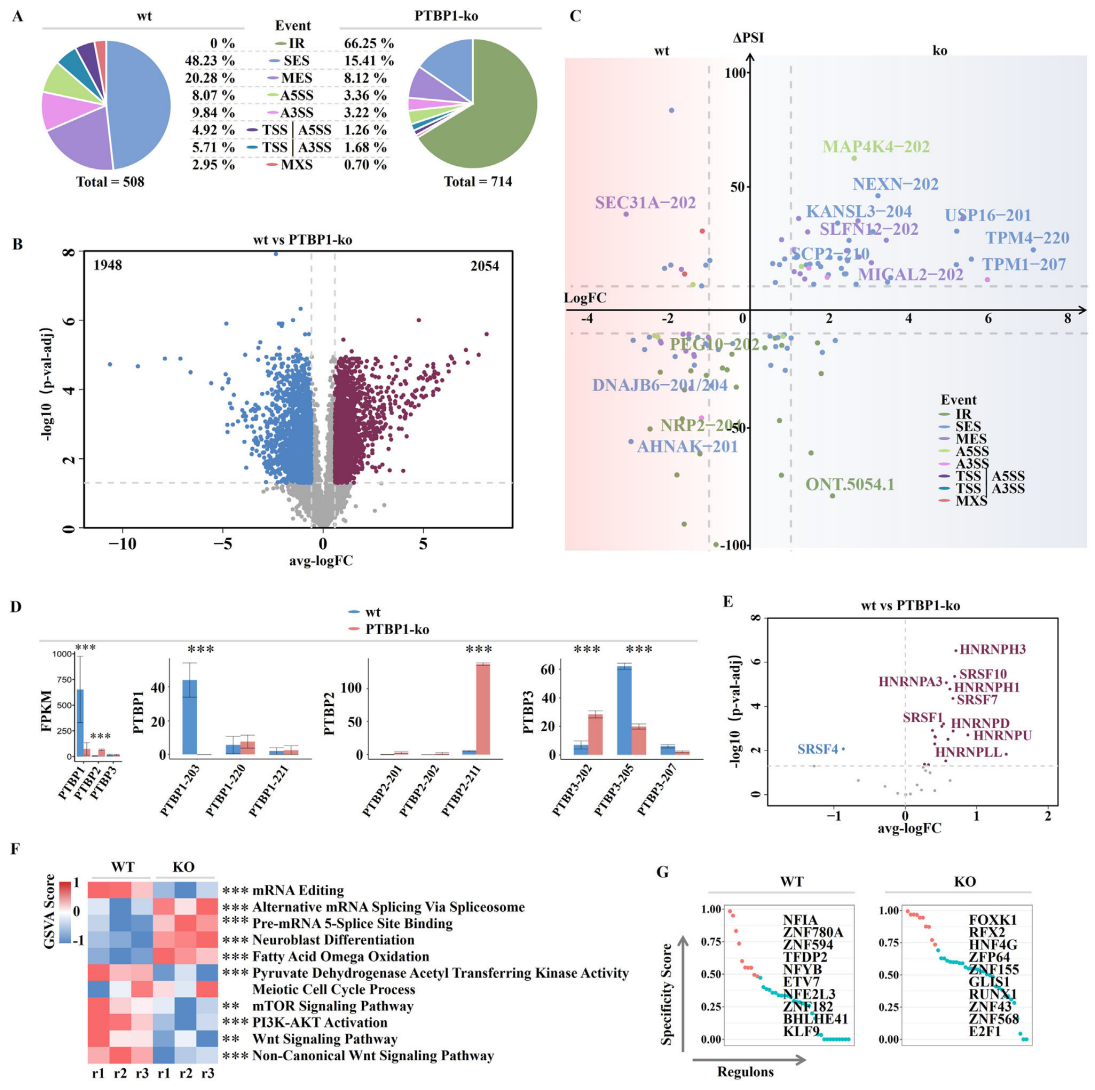
PTBP1 regulates alternative splicing in ccRCCs. (A) Pie charts of alternative splicing events in wild type and PTBP1 knockout cells. (B) Volcano plot of differentially expressed genes between wild type and PTBP1 knockout cells (red: upregulated in PTBP‐ko group). (C) Joint analysis of differentially expressed transcripts and splicing events, with the x‐axis representing the fold difference of transcripts and the y‐axis representing the ΔPSI value of splicing events. (D) All PTBP members and their transcript variants expression in wild type and PTBP1 knockout cells. (E) Volcano plot of differentially expressed splicing factors between wild type and PTBP1 knockout cells. (F) Heatmap of GSVA score of main pathways. (G) Analysis of the specific transcription factor activity in the PTBP1‐KO group and the WT group. Statistical tests involved: *P < 0.05, **P < 0.01, ***P < 0.001, Student's t‐test; Data are expressed as mean ± SD, *n* = 3.

Given that PTBP1 functions as an alternative splicing factor, we employed full‐length transcriptome sequencing to obtain comprehensive information on splicing events. Splicing events (Figure 3A) and differentially expressed genes (Figure 3B) were further analyzed between WT and KO cells. Then, we analyzed splicing events by ∆PSI and log FC (Figure 3C). Knowing that PTBP1 has two family members, PTBP2 and PTBP3, we wondered whether there was functional redundancy among members of the PTBP family. Results in Figure 3D showed that, PTBP1 is dominant in RCC cells compared to the other two members, especially PTBP1‐203 variants. However, an increase in other splicing factors, such as HNRNPH3, HNRNPH1 and SRSF10 was observed following PTBP1 knockout, indicative of a compensatory effect (Figure 3E). Pathway enrichment analysis showed that WT group had higher score of PI3K/AKT activation and mTOR signaling pathway compared to PTBP1‐KO group (Figure 3F). Moreover, regulon analysis revealed a global shift in transcription factor activity induced by PTBP1 depletion (Figure 3G). Therefore, we found that PTBP1 indeed significantly influenced AKT/mTOR pathway activity, although the precise regulatory mechanism remains to be elucidated.

### 2.4 PTBP1 Regulates Alternative Splicing of DNAJB6 mRNA

**FIGURE 4:**
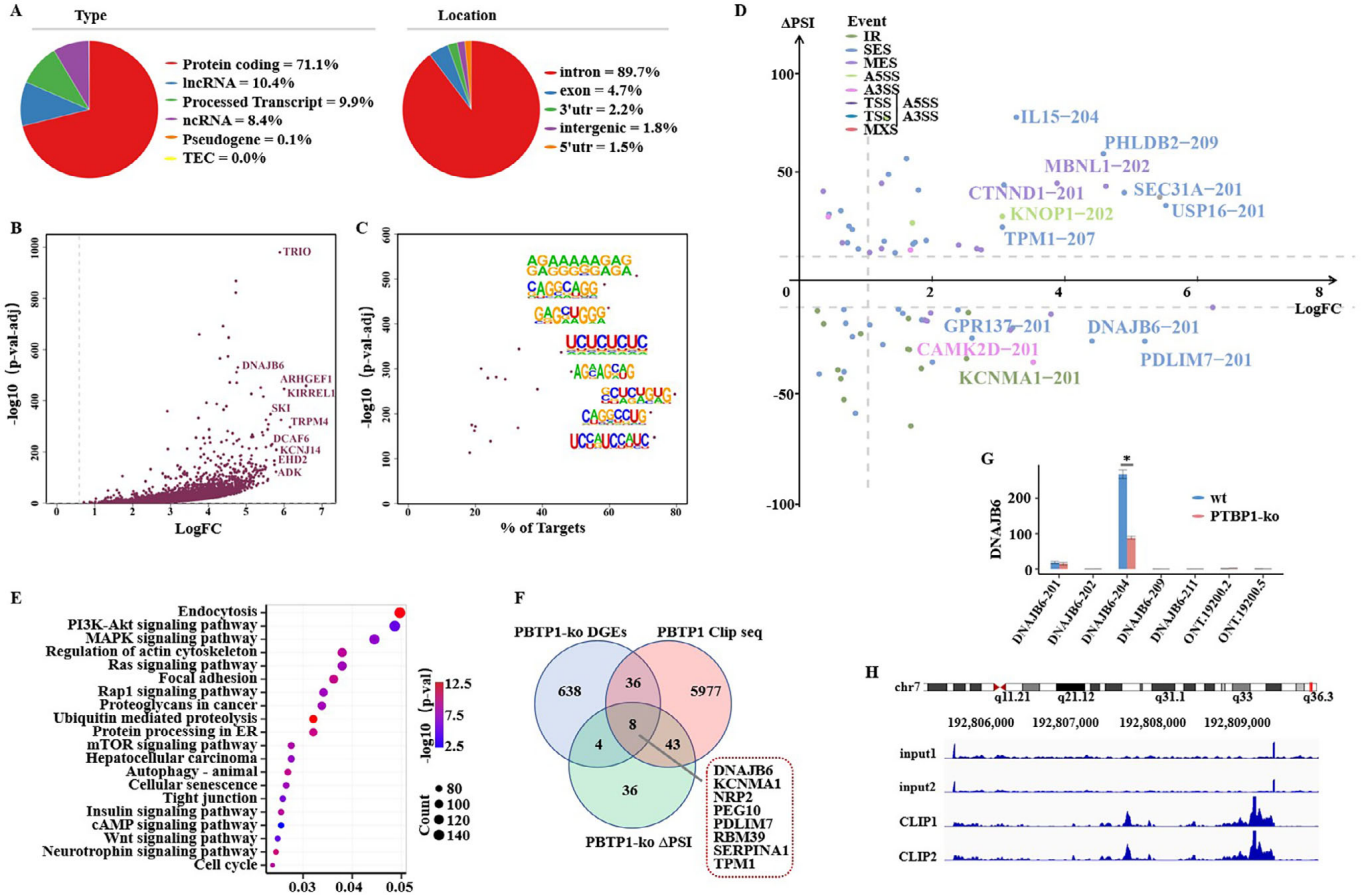
PTBP1 regulates alternative splicing of DNAJB6 mRNA. (A) Pie charts of PTBP1‐binding mRNA and location. (B) Volcano of significantly enriched mRNA transcripts by CLIP. (C) Main binding motif of PTBP1. (D) Joint analysis of CLIP‐seq binding peaks and full‐length seq variable splicing, with the x‐axis representing the fold difference in binding peak abundance and the y‐axis representing the ΔPSI value of the splicing event. (E) Pathway enrichment analysis of the PTBP1‐bound transcripts. (F) CLIP‐seq, full‐length‐seq differential alternative splicing, and full‐length‐seq differentially expressed genes jointly screen the key targets regulated by PTBP1. (G) Expression differences of the DNAJB6 transcript between the PTBP1‐KO group and the WT group. (H) Analysis of the binding peak positions of PTBP1 on DNAJB6 pre‐mRNA. Statistical tests involved: *P < 0.05, **P < 0.01, ***P < 0.001, Student's t‐test; Data are expressed as mean ± SD, *n* = 3.

PTBP1 was already known as an RNA binding protein (RBP). It recognizes and binds to poly‐pyrimidine sequences of pre‐mRNA through RNA recognition motifs (RRMs). We performed cross‐link immunoprecipitation (CLIP) sequencing to identify the potential downstream genes of PTBP1. PTBP1 mainly regulates the alternative splicing of protein‐coding mRNA and binds to introns (Figure 4A). mRNA enriched by CLIP was shown in Figure 4B, and the motifs that PTBP1 recognized were UC‐rich regions (Figure 4C). We further performed an integrative analysis on ∆PSI and binding abundance (Figure 4D). The results indicated that, overall, transcripts with stronger PTBP1 binding signals showed a higher tendency to undergo significant splicing pattern changes upon PTBP1 loss. Besides, the pathway involved in gene enriched by CLIP included endocytosis and PI3K/AKT signaling pathway (Figure 4E). Furthermore, we integrated the results of PTBP1‐KO DEGs, PTBP1‐KO ∆PSI, PTBP1 CLIP‐seq, and then identified 8 candidates (Figure 4F). Among them, we noticed DNAJB6. Previous reports on the role of DNAJB6 in cancer have shown inconsistencies. DNAJB6 belongs to heat shock protein 40 (Hsp40) family, including 2 main transcripts variants, DNAJB6a (DNAJB6‐201, NM_058246.4) and DNAJB6b (DNAJB6‐204, NM_005494.3). The short isoform DNAJB6b was the predominant isoform in ccRCC cells compared to the longer one (Figure 4G). We speculated that these two isoforms exert opposite effects. Peak scanning showed PTBP1 mainly binds to the 3’ terminal region of DNAJB6 pre‐mRNA, which led to splicing of the terminal region and generation of the short isoform. (Figure 4H).

### 2.5 DNAJB6b Expression is Promoted by PTBP1 and Influences EVE Sensitivity

**FIGURE 5:**
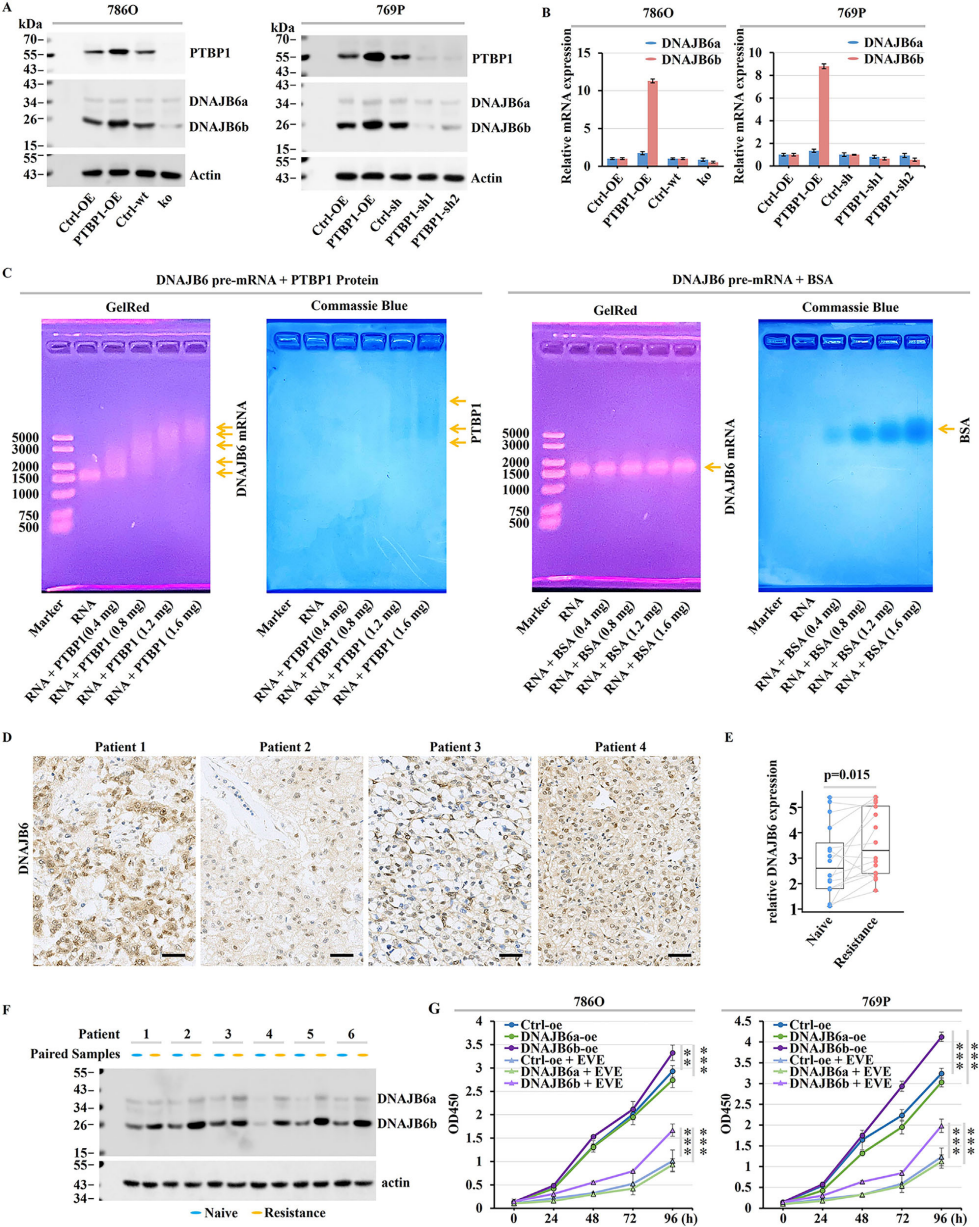
DNAJB6b expression is promoted by PTBP1 and influences EVE sensitivity. Western blotting (A) and RT‐qPCR (B) detecting the protein expression of DNAJB6a and DNAJB6b in the PTBP1 overexpression (OE), knockout (KO), or knockdown (sh) groups. (C) Electrophoretic mobility shift assay detecting the direct binding of PTBP1 to DNAJB6 pre‐mRNA. (D) Representative images of immunohistochemical staining of DNAJB6 in ccRCC tissues from patients resistant to EVE, scale bar = 50 µm. DNAJB6 was upregulated after resistance to EVE developed compared to before treatment, which was assessed by western blotting (E) and immunohistochemical staining (F). (G) Overexpression of DNAJB6b, but not DNAJB6a, could weaken the proliferation rate of ccRCC cells, and could cooperate with EVE treatment. Statistical tests involved: *P < 0.05, **P < 0.01, ***P < 0.001, Student's t‐test; Data are expressed as mean ± SD, *n* = 3.

We further performed RT‐qPCR and western blot assay. The results showed that knockout or knockdown of PTBP1 decreased the expression of DNAJB6b mRNA and protein (Figure 5A,B, Figure S4A). Besides, Re‐expression of DNAJB6b effectively rescued the AKT/mTOR pathway inhibition resulting from PTBP1 depletion (Figure S4B). We subsequently performed electrophoretic mobility shift assay. A shifted band of DNAJB6 pre‐mRNA indicated a direct interaction with the PTBP1 protein (Figure 5C, left), whereas the shift did not exhibit in pre‐mRNA and BSA group (Figure 5C, right). Collectively, our findings established that PTBP1 controls DNAJB6 alternative splicing by binding to its pre‐mRNA directly.

Since immunohistochemistry cannot distinguish between the DNAJB6a and DNAJB6b isoforms, and given that DNAJB6b is the predominant form, we used total DNAJB6 staining as a surrogate for DNAJB6b expression. We observed DNAJB6b was highly expressed in EVE‐resistant tumors (Figure 5D). An increase in DNAJB6b expression was observed in patients after they acquired resistance to EVE, compared to before treatment (Figure 5E). Besides, western blotting assay also showed DNAJB6b significantly increased after resistance developed (Figure 5F). Additionally, we found DNAJB6b, instead of DNAJB6a, would influence the sensitivity to EVE (Figure 5G, Figure S5).

### 2.6 DNAJB6b Interacts With EIF4B

**FIGURE 6:**
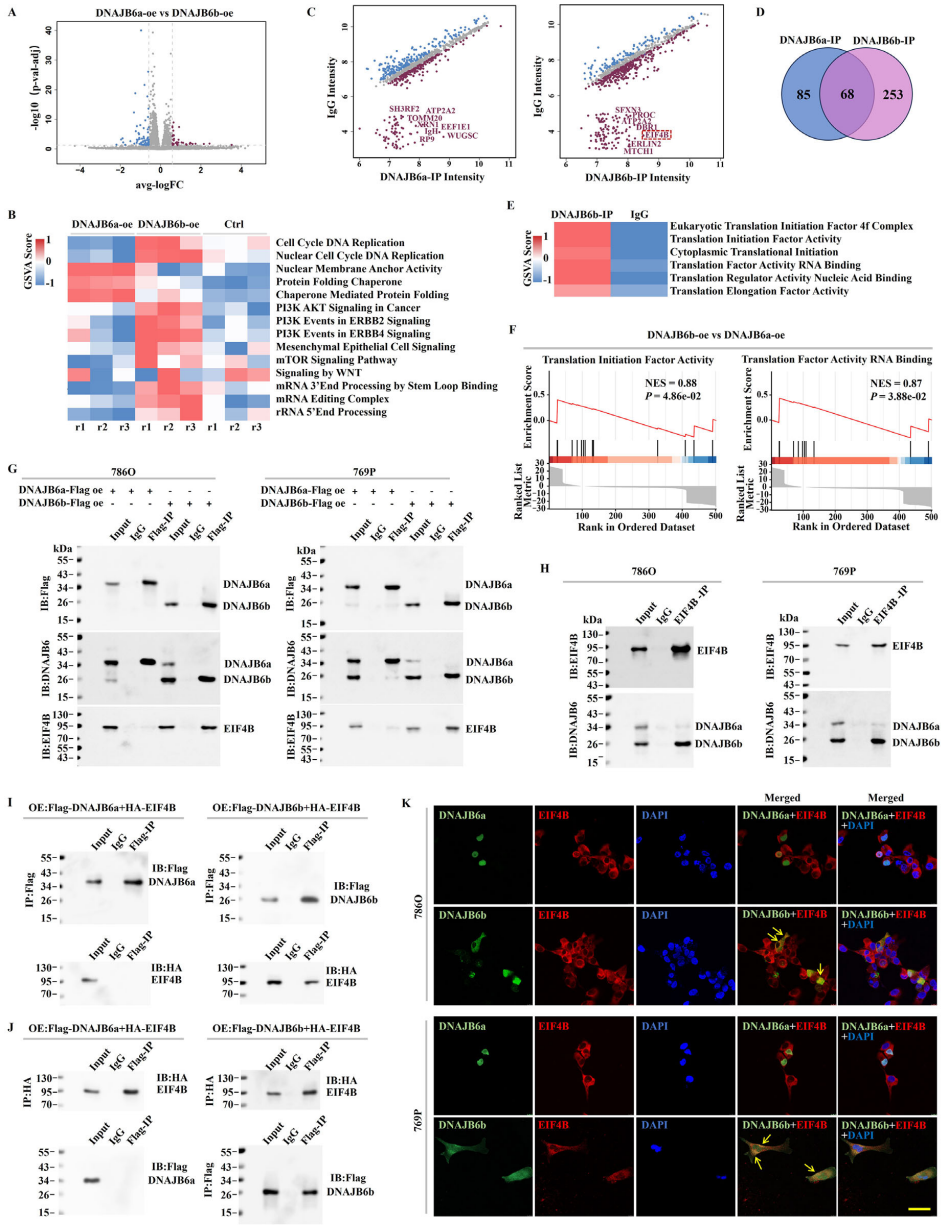
DNAJB6b interacts with EIF4B. (A) Analysis of differentially expressed genes in the transcriptome of 293T cells after overexpression of DNAJB6a and DNAJB6b. (B) GSVA pathway analysis of the DNAJB6a‐oe group, DNAJB6b‐oe group, and ctrl group (*n* = 3 per group). (C) The bound proteins of DNAJB6a (left) and DNAJB6b (right), with the x‐axis representing the binding intensity of the IP group and the y‐axis representing the binding intensity of the control IgG group. (D) IP‐MS shows that there are differences in the protein spectra of DNAJB6b and DNAJB6a binding proteins. (E) GSVA pathway analysis of the bound proteins in the DNAJB6b‐IP group and the control IgG group. (F) GSVA pathway analysis of the differentially bound proteins between the DNAJB6b‐IP group and the DNAJB6a‐IP group. (G) Flag‐tagged targeted immunoprecipitation assay showed EIF4B bound to DNAJB6b but not DNAJB6a. (H) EIF4B targeted immunoprecipitation assay showed DNAJB6b but not DNAJB6a bound to EIF4B. Dual‐tagged co‐immunoprecipitation assay which is incubated with anti‐flag beads (I) or anti‐HA beads (J) to detected the interaction between FLAG‐tagged DNAJB6a/b and HA‐tagged EIF4B. (K) Immunofluorescence assay to detected the subcellular localization of DNAJB6a/b and EIF4B.

Flag‐tagged DNAJB6a and DNAJB6b were respectively overexpressed and then transcriptome sequencing was performed (Figure 6A). Compared to DNAJB6a‐oe and control group, DNAJB6b‐oe group showed higher score of AKT/mTOR associated‐ and mRNA editing complex pathway (Figure 6B). As DNAJB6 acts as a molecular chaperone, whose role in transcriptional regulation might not be prominent, we sought to investigate its interacting proteins to elucidated its biologic function. Accordingly, flag‐tagged DNAJB6a and DNAJB6b overexpressed lysate were mixed with anti‐flag beads or IgG beads, and then used for mass spectrometry analysis (Figure 6C). The results of IP‐MS showed that proteins co‐immunoprecipitated solely by DNAJB6b were more abundant than by DNAJB6a (Figure 6D), which may indicate more complicated function assumed by DNAJB6b complex, especially translation regulation (Figure 6E,F). Eukaryotic Translation Initiation Factor 4B (EIF4B), a known effector of the mTOR signaling whose Ser422 is phosphorylated by protein kinase S6K [26, 27], was identified as a significant interactor of DNAJB6b (Figure 6C, right). Then, we performed flag‐tagged‐DNAJB6a/b targeted immunoprecipitation to confirm the interaction between DNAJB6b and EIF4B, whereas this interaction was not observed between DNAJB6a and EIF4B (Figure 6G). Similarly, same results were shown in EIF4B targeted immunoprecipitation (Figure 6H). Additionally, dual‐tagged co‐immunoprecipitation assay (Figure 6I,J) and GST‐pulldown assay (Figure S6) further validated this isoform‐specific interaction between DNAJB6b and EIF4B. Moreover, we also investigated the subcellular localization of DNAB6a/b. The results showed that DNAJB6a mainly localized in nucleus while DNAJB6b in cytoplasm (Figure 6K). Since EIF4B‐mediated translation occurs in the cytoplasm, it is reasonable to propose that DNAJB6b is the principal isoform that interacts with it.

### 2.7 DNAJB6b Binds to EIF4B and Activates Oncogenic Translation

**FIGURE 7:**
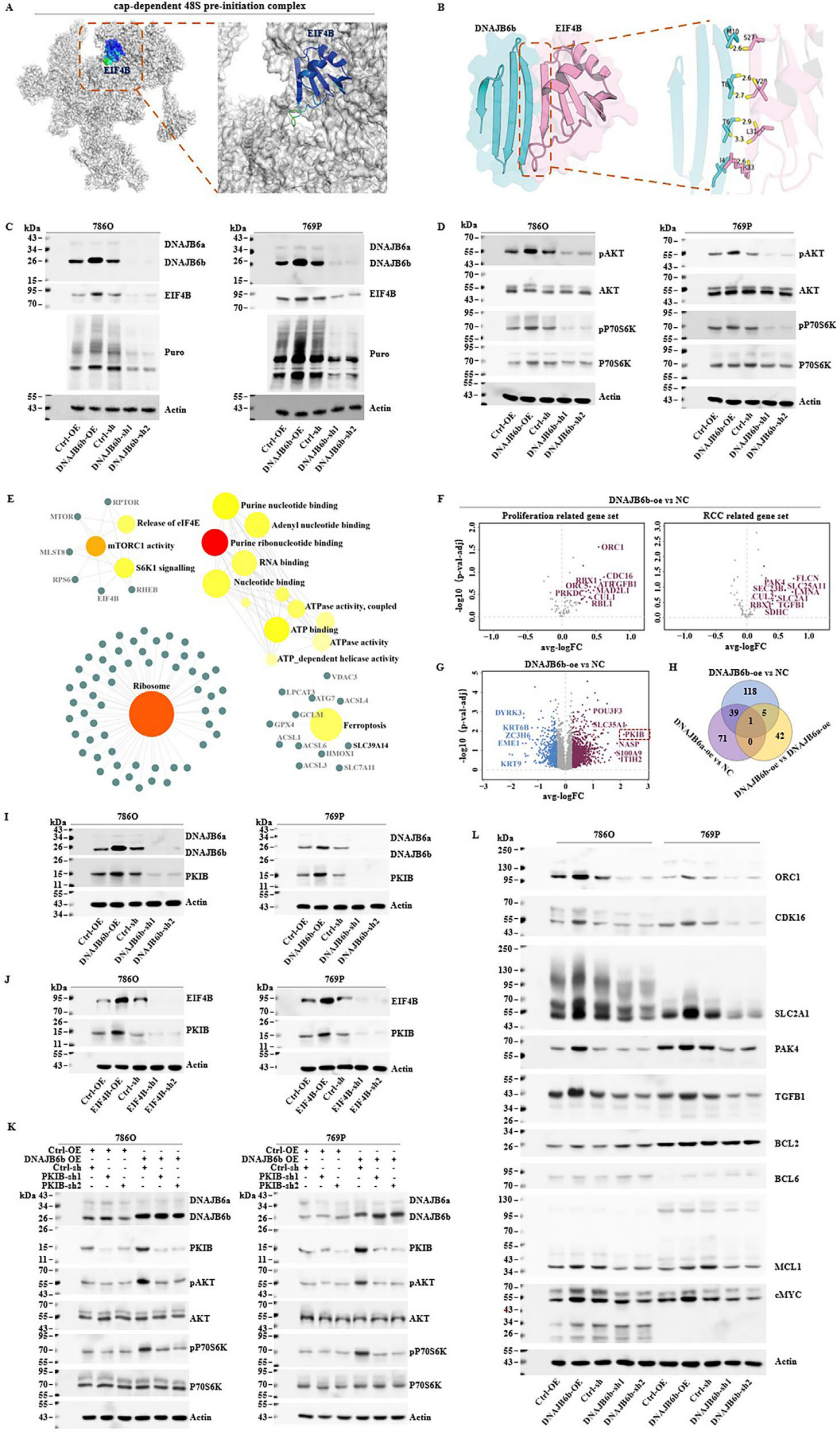
DNAJB6b bounds to EIF4B and activates PKIB/AKT/mTOR feedback loop. (A) Structure of EIF4B within the cap‐dependent 48S pre‐initiation complex. (B) AlphaFold‐Multimer prediction of the DNAJB6b CTD‐EIF4B RRM complex. The top‐ranked model (rank_1) is shown, with DNAJB6b CTD colored in cyan and EIF4B RRM in pink. A close‐up view highlights predicted hydrogen bonds and hydrophobic contacts at the interface. (C) Effect of overexpression of DNAJB6b (DNAJB6b‐oe) and knockdown of DNAJB6b (DNAJB6b‐sh) on protein translation efficiency assessed by SUnSET assay. (D) Western blotting analysis of the effects of overexpression (DNAJB6b‐oe) and knockdown (DNAJB6b‐sh) of DNAJB6b on the phosphorylation levels of AKT and P70S6K. (E) PPI network analysis of differentially expressed proteins between the DNAJB6b overexpression group (DNAJB6b‐oe) and the control group (nc). (F) Analysis of differentially expressed proteins in the proliferation (left) and RCC‐related (right) gene sets between the DNAJB6b‐oe group and the NC group (red: upregulated in DNAJB6b‐oe group). (G) Total analysis of differentially expressed proteins between the DNAJB6b‐oe group and the NC group. (H) Differences in protein expression profiles among the DNAJB6b‐oe, DNAJB6a‐oe groups and the NC group (*n* = 3 per group). (I) Western blotting detecting the effect of overexpression of DNAJB6b (DNAJB6b‐oe) and knockdown of DNAJB6b (DNAJB6b‐sh) on the abundance of PKIB protein. (J) Western blot detecting the effect of overexpression of EIF4B (EIF4B‐oe) and knockdown of EIF4B (EIF4B‐sh) on the abundance of PKIB protein. (K) Western blot analysis of the effect of PKIB knockdown (PKIB‐sh) on the phosphorylation of AKT and P70S6K induced by overexpression of DNAJB6b. (L) Western blotting analysis of the effects of overexpression of DNAJB6b (DNAJB6b‐oe) and knockdown of DNAJB6b (DNAJB6b‐sh) on the protein abundances of ORC1, CDK16, SLC2A1, PAK4, TGFB1, BCL2, BCL6, MCL1, and cMYC.

EIF4B is a pivotal regulatory factor in eukaryotic translation initiation [28, 29]. It functions as an RNA‐binding protein and a scaffold that dynamically interacts with both the ribosome and the core translation initiation complex (notably the EIF4F complex) to facilitate the assembly of the 48S pre‐initiation complex and promote the scanning and attachment of mRNA to the ribosome (Figure 7A). Next, virtual molecular docking was performed to predict the potential complex formed between the C‐terminal domain (CTD) of DNAJB6b and the RNA recognize motif domain of EIF4B. Among the five predicted models generated, the top‐ranked model exhibited high confidence (Figure 7B and Figure S7A,B). This result strongly suggested that DNAJB6b might directly anchor onto the functional domain of EIF4B via its unique CTD, thereby exerting an influence on the activity or stability of EIF4B.

As anticipated, overexpression of DNAJB6b significantly increased global protein biosynthesis, whereas knockdown of DNAJB6b decreased it (Figure 7C, Figure S8A). Besides, overexpression of DNAJB6b led to increasingly activated mTOR signaling while knockout or knockdown of it would inhibit the signaling (Figure 7D, Figure S8B). Since EIF4B mainly influenced the protein translation, we performed proteome sequencing analysis. PPI analysis between DNAJB6b‐oe cells and control group focused on several significant functions such as mTORC1 activity, ribosome and purine ribonucleotide binding (Figure 7E). Besides, proliferation related genes and RCC related genes were significantly enriched in DNAJB6b‐oe group compared to control group (Figure 7F). Thus, DNAJB6b interacts with EIF4B to form a complex that functions as a downstream effector, thereby further potentiating mTOR‐driven oncogenic translation.

### 2.8 DNAJB6b/EIF4B Upregulates PKIB and Forms a Positive Activation Loop

We had already found DNAJB6b contributes to amplifying mTOR signaling. However, given its position as a downstream component, this mechanism was insufficient to fully account for the EVE resistance. We further investigated all differential expression proteins between DNAJB6b‐oe group and control group and we noticed PKIB upregulated in DNAJB6b‐oe group (Figure 7G,H), which played an essential role in AKT activation [30, 31, 32]. PKIB expression shows a positive correlation with DNAJB6b (Figure 7I, Figure S8C). Furthermore, a significant positive correlation is also observed between PKIB and EIF4B (Figure 7J, Figure S8D). DNAJB6b overexpression promoted AKT/P70S6K activation and EVE resistance, both of which were abrogated by PKIB knockdown (Figure 7K, Figure S9A). Moreover, DNAJB6b overexpression significantly upregulated the translation of oncogenic proteins, such as c‐MYC, MCL1, BCL2 and CDK2 (Figure 7L), underscoring its potent tumor‐promoting capability. Above all, our study shows that DNAJB6b associates with EIF4B and drives PKIB expression, resulting in both amplification of downstream mTOR signaling and persistent activation of upstream AKT, which collectively leads to the development of EVE resistance. This signaling pathway was also corroborated in patient‐derived samples (Figure S9B).

### 2.9 Inhibition of PTBP1 Enhances the Sensitivity to EVE In Vivo

**FIGURE 8:**
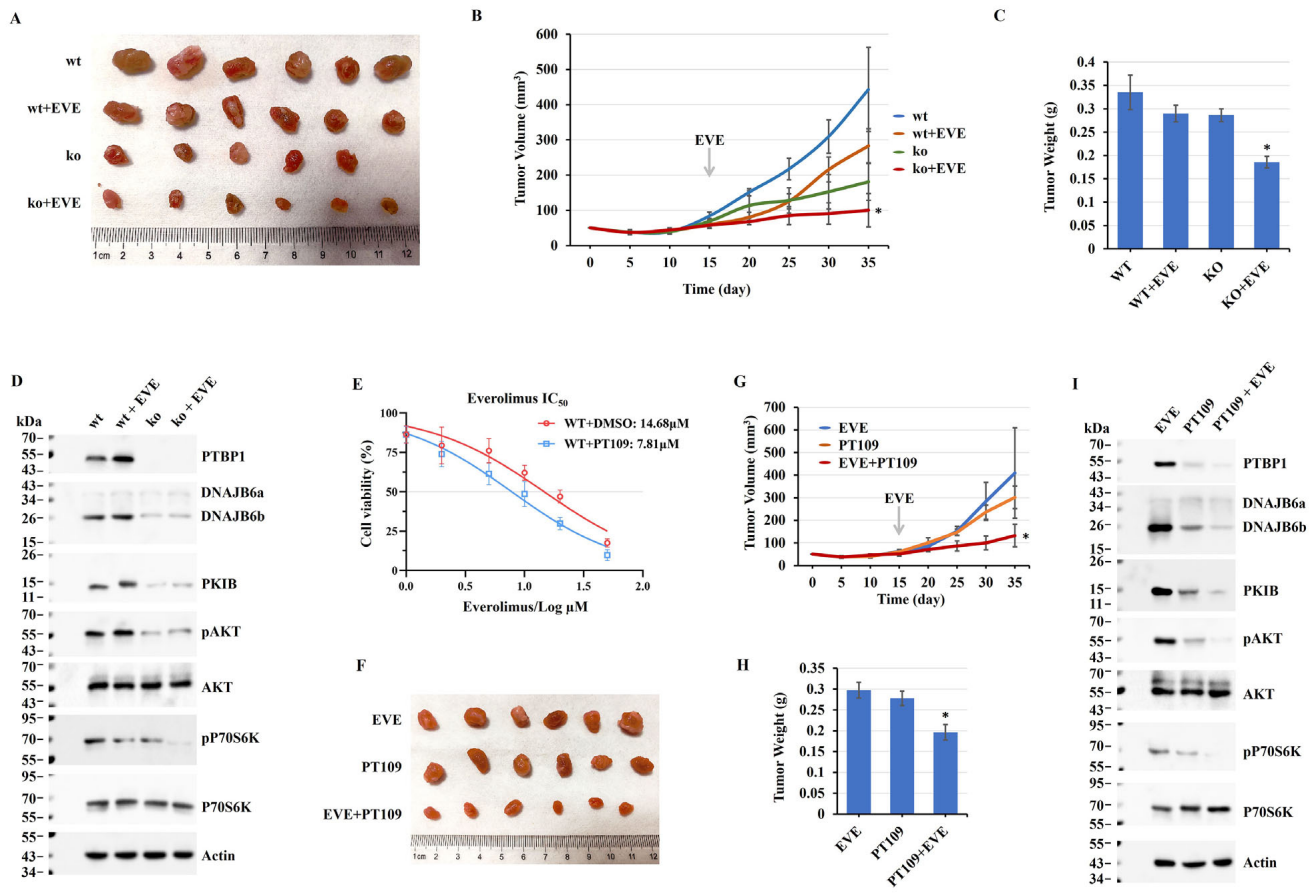
Inhibition of PTBP1 enhances the sensitivity to EVE in vivo. (A) Tumors harvested from EVE‐treatment or placebo group. (B) Tumor growth curves of 4 group. (C) The weights of the four groups of harvested tumors. (D) Western blotting results of 4 group tumors. (E) PT109 increased the sensitivity to EVE treatment. (F) Tumors harvested from EVE‐treatment, PT109‐treatment, and placebo group. (G) Tumor growth curves of 3 group. (H) The weights of the 3 groups of harvested tumors. (I) Western blotting results of 3 group tumors. Statistical tests involved: *P < 0.05, **P < 0.01, ***P < 0.001, Student's *t*‐test; Data are expressed as mean ± SD, *n* = 3.

We further explored the impact of PTBP1 intervention on EVE efficacy in an in vivo model. Knockout of PTBP1 not only inhibited the growth of tumor, but also could enhance the antitumor effect of EVE (Figure 8A–C). Mechanistically, in the EVE monotherapy group, despite the inhibition of P70S6K phosphorylation, AKT was consequently activated via negative feedback (Figure 8D, Figure S10A). Notably, PTBP1 knockout suppressed this negative feedback activation and further enhanced the efficacy of EVE treatment (Figure 8D, Figure S9A). Moreover, we used a PTBP1 inhibitor to validate this therapeutic effect. PT109 was a small molecular inhibitor of several kinases, also recognized as a PTBP1 silencer [33]. PT109 could enhance the sensitivity of ccRCC cells to EVE in vitro (Figure 8E). Consistently, PT109 could also block the feedback activation and cooperate with EVE to suppress tumor growth (Figure 8F–H). Western blot analysis of tumor tissues further revealed that the combination of PT109 and EVE led to maximal inhibition of the AKT/mTOR signaling (Figure 8I, Figure S10B). Above all, targeting PTBP1 might sever as a strategy against EVE resistant and offer promise for patients with advanced ccRCCs.

## 3 Discussion

It is widely recognized that alternative splicing abnormalities can produce aberrant proteins that lead to various aspects of tumorigenesis, including proliferation, invasion, metastasis, angiogenesis, and drug resistance. Trans‐acting splicing factors are a class of alternative splicing‐associated RBPs that recognize and bind to cis‐regulatory elements on pre‐mRNA, affecting the exclusion of exons or retention of introns. Dysfunction of these splicing factors results in aberrant AS events in tumors, which further promote tumor proliferation, angiogenesis, immunosuppression and therapy resistance. Alternative splicing factor mainly concludes ubiquitously expressed heterogeneous nuclear ribonucleoproteins (hnRNPs) and the serine/arginine (SR)‐rich family of pre‐mRNA splicing factors (SRSFs). PTBP1, belonging to the former, recognizes and binds to the polypyrimidine tract of introns and promote the binding of U2 snRNP to pre‐mRNA. Wang et al. have reported that PTBP1 promotes the growth of breast cancer cells through PTEN/AKT pathway [24]. Besides, PTBP1‐mediated skipping of exon 10 in Axl pre‐mRNA promotes phosphorylation of AKT protein and contributes to metastasis of liver cancer cells [21]. Our study observed that PTBP1 was correlated with AKT/mTOR signaling pathway though bioinformatic analysis. In patients and mice tumor specimens, PTBP1 was found upregulated in EVE‐resistant tissues, which highlighted its potential as a biomarker and a therapeutic target.

Mechanistically, we integrated multi‐omics analyses to elucidate the molecular mechanisms by which PTBP1 regulates alternative splicing. DNAJB6 was identified as a vital downstream of PTBP1 with its last exon of pre‐mRNA altered and therefore activated AKT/mTOR pathway to promote progression and resistance of ccRCC. DNAJB6, also known as MRJ, is a member of heat‐shock proteins 40 (Hsp40) family, which is responsible for interacting with the ATPase domain, and possibly the substrate‐binding domain of its partner Hsp70. There are two main spliced isoforms of human DNAJB6; DNAJB6a, a 326 aa protein coded by DNAJB6‐201(long isoform, 2.5 kb transcript variant I, NM_058246) and DNAJB6b, a 241 aa protein coded by DNAJB6‐204 (short isoform, 1.6 kb transcript variant II, NM_005494). The short isoform protein lacks the carboxyl‐terminal 95 amino acids compared to the large form but contains an additional 10 amino acids (KEQLLRLDNK). In previous research, DNAJB6 exhibited context‐dependent functions across various tumor types, a phenomenon potentially driven by distinct isoform expression patterns [34, 35, 36, 37, 38, 39, 40]. The long isoform has a C‐terminal nuclear localization sequence (NLS). Its nuclear localization is associated with survival of patients with esophageal cancer and reduces AKT signaling [35]. On the contrary, the short isoform lacks the NLS, is located in cytoplasm, and may be associated with malignant behavior in cancer. In previous reports, few studies have focused exclusively on the short isoform, whereas most have studied both isoforms as an integrated entity. In colorectal cancer, overexpression of DNAJB6 promotes cancer cell invasion through IQGAP/ERK‐dependent pathway [40]. Jiang et al. also developed a prognostic prediction model for CRC with three proteins including DNAJB6 [41]. The short isoform was found to be predominant in the above studies, a pattern that was also observed in our investigation of ccRCC. Interestingly, though lacking of NLS, DNAJB6b was reported to migrate into nucleus under cellular stress like heat shock or hypoxia. Andrew et al. reported that hypoxia drove DNAJB6b to the nucleus, and therefore promotes the proliferation and invasiveness of tumor cells [42]. In context of the loss of VHL and hyperactivation of HIF in ccRCC, we hypothesized that DNAJB6b might participate in other important signaling pathways. In summary, our study focuses on DNAJB6b, as a downstream target of PTBP1‐mediated alternative splicing, thereby filling a gap in previous reports on DNAJB6 and enriching the understanding of its biology.

In‐depth investigation of DNAJB6b revealed its crucial partner, EIF4B, and another key mediator PKIB. EIF4B could be regulated and phosphorylated by proto‐oncogenic signaling pathways [26, 43], and then promoted oncogenic translation [44, 45]. Drug targeting EIF4B could inhibit tumor growth via AKT/mTOR signaling [46]. As DNAJB6b is robustly produced via PTBP1‐mediated alternative splicing, its interaction with the RNA‐binding domain of EIF4B facilitates oncogenic translation. Intriguingly, PKIB expression increased concomitantly with DNAJB6b overexpression, forming a positive feedback loop that consistently activated the AKT/mTOR signaling pathway. These findings recapitulated the well‐recognized mechanism underlying EVE resistance, namely that mTOR inhibition triggers negative feedback activation of upstream AKT [47, 48]. Moreover, we found that inhibition of PTBP1/DNAJB6b suppressed tumor growth and, more importantly, sensitized tumors to EVE.

We acknowledged several limitations in this study. First, although we identified a strong correlation between PTBP1 upregulation and acquired EVE resistance, the upstream signals linking mTOR inhibition to increased PTBP1 expression remain to be elucidated. Second, given the widespread use of combination regimens comprising immune checkpoint inhibitors (ICIs) and tyrosine kinase inhibitors in ccRCC, the crosstalk between mTOR inhibitor and these drug classes warrants further investigation in immunocompetent models. Notably, previous studies have documented the immunostimulatory properties of mTOR inhibitors and their synergy with ICIs [49, 50, 51, 52]. Therefore, overcoming EVE resistance may support the development of EVE–ICI combination regimens, which represents one of our future directions.

## 4 Conclusions

Collectively, our findings pinpointed the PTBP1/DNAJB6b/EIF4B/PKIB axis as a key mediator of EVE resistance. Therefore, targeting this axis offers a promising therapeutic avenue to restore EVE sensitivity in patients with advanced ccRCCs.

## 5 Methods

### 5.1 Single‐Cell Transcriptome Sequencing (scRNA‐seq)

Tissues were collected, rinsed, minced, and dissociated using SeekMate Kits at 37°C with DNase I added as needed. The suspension was filtered, lysed for RBCs, and assessed for viability. Viable samples (> 85%) were debris‐purified, if necessary, and resuspended in RPMI‐1640 with 2% FBS at 1 × 10^6 cells/ml. Single‐cell libraries were prepared using a SeekOne 3' kit. Single cells were mixed with RT reagents and partitioned into droplets with barcoded beads. RT was performed (42°C, 90 min; 85°C, 5 min). After droplet breakage, cDNA was purified, amplified, fragmented, end‐repaired, A‐tailed, adaptor‐ligated, and index‐PCR amplified. Libraries were purified and quality‐checked. Sequencing was done on a SURFSeq Q (PE150). FASTQ reads (≥90% Q30) were demultiplexed, processed for barcode/UMI counting, and aligned to GRCh38. The resulting matrix was used for downstream analysis. Single‐nucleus RNA‐seq data were obtained from the National Center for Biotechnology Information Gene Expression Omnibus (accession no. GSE240822, GSE227898, GSE151302, GSE183277).

Data were analyzed with Seurat. snRNA‐seq nuclei: < 200 or > 10 000 genes, > 10 000 UMIs, or > 10% mitochondrial reads excluded. scRNA‐seq cells: < 200 or > 6000 genes, > 10 000 UMIs, > 25% mitochondrial or > 5% hemoglobin reads excluded. Genes detected in < 3 cells removed. Doublets were removed. Top 2000 variable genes and first 30 PCs were used for clustering with Harmony batch correction. UMAP and subclustering followed the same workflow.

### 5.2 Transcriptome Sequencing

Total RNA was extracted from cultured cells, and assessed for purity, concentration, and integrity using advanced instrumentation. For full‐length sequencing, sequencing libraries were constructed following the standard protocol from Oxford Nanopore Technologies (ONT). Briefly, poly(A)+ RNA was reverse‐transcribed into full‐length cDNA using template‐switching oligos. Following second‐strand synthesis, DNA damage repair and end repair were performed, and the products were purified using magnetic beads. Sequencing adapters were then ligated to the cDNA fragments. Prepared libraries were loaded onto an ONT flow cell and sequenced on a PromethION or GridION platform using real‐time single‐molecule sequencing. Base‐calling was performed using Guppy within the MinKNOW software suite, converting raw current signals (fast5) into nucleotide sequences (fastq). Sequencing quality was assessed, and reads shorter than 500 bp or with a mean Q‐score below 6 were filtered out, along with ribosomal RNA sequences, to obtain clean reads for downstream bioinformatics analysis. For second‐generation transcriptome, the RNA libraries were constructed on the illumina NovaseqTM 6000 platform by Bioprofile technology (Shanghai, China).

### 5.3 Cross‐Link Immunoprecipitation (CLIP) Sequencing

CLIP was performed to identify genome‐wide RNA binding sites for PTBP1. Briefly, cells (5 × 10^6^) were crosslinked with 1% formaldehyde for 10 min, followed by quenching with 0.125 M glycine. After washing with PBS, cells were collected and lysed in IP buffer supplemented with protease and RNase inhibitors, followed by sonication. A portion of lysate was saved as input. Lysates were incubated overnight with anti‐PTBP1 antibody conjugated to Protein A/G magnetic beads. Immunoprecipitated RNA‐protein complexes were washed, treated with proteinase K to reverse crosslinks, and RNA was extracted using TRIzol. Libraries were prepared from RNA by reverse transcription, end repair, A‐tailing, adapter ligation, and PCR amplification, followed by sequencing on an Illumina NovaSeq 6000 platform.

Raw reads were processed using fastp to remove adapters and low‐quality bases. rRNA sequences were filtered with Bowtie2. Clean reads were aligned to the reference genome (hg38) using HISAT2. PCR duplicates were removed with Picard. Peaks were called using MACS2 and annotated with BedTools. Motif analysis was performed with HOMER. Functional enrichment analysis of peak‐associated genes was conducted using clusterProfiler. Transcript expression and differential expression were analyzed with StringTie and DEGseq/edgeR, respectively.

### 5.4 Protein Immunoprecipitation

Cells were transfected with plasmid for 48 h and then lysed. Specific beads were incubated with lysate for 2 h at room temperature. Then beads were separated, washed and save at −80°C for further mass spectrometry analysis, or boiled with SDS loading buffer for western blotting assay.

### 5.5 Mass Spectrometry and DIA Proteomic

Mass spectrometry and DIA proteomic was performed by Bioprofile technology (Shanghai, China). Cell samples were harvested, pelleted by centrifugation and then added SDS buffer. Samples were reduced with DTT, alkylated with IAA, and digested with trypsin at 37°C overnight. The resulting peptides were desalted on an MCX column, vacuum‐concentrated, and reconstituted in formic acid. Peptide concentration was determined by UV absorbance at 280 nm. For DIA analysis, iRT calibration peptides were added prior to analysis. Peptides were analyzed on an Orbitrap Astral mass spectrometer coupled to a Vanquish Neo LC system (Thermo Scientific) using DIA mode. Precursor scanning: 380–980 m/z; MS1 resolution: 240 000 at 200 m/z; AGC target: 500%; max IT: 5 ms. MS2 DIA employed 299 windows (2 m/z isolation), HCD collision energy of 25 eV, AGC target of 500%, and max IT of 3 ms. DIA data were analyzed with DIA‐NN 1.8.1.

### 5.6 AlphaFold‐Multimer‐Based Predictions of Protein Structure

The structure of the DNAJB6b–EIF4B complex was predicted using AlphaFold‐Multimer implemented in ColabFold (v1.6.1) with default parameters. Amino acid sequences corresponding to the indicated domains were used as input for prediction. Specifically, the C‐terminal domain (CTD) of DNAJB6b spanning residues Phe189 to Ile229 and the RNA recognition motif (RRM) domain of EIF4B spanning residues Tyr96 to Gln173 were used for complex prediction. The predicted complex structures were evaluated using the predicted aligned error (PAE) and predicted local distance difference test (pLDDT) scores. The top‐ranked model was visualized and analyzed using PyMOL (Schrödinger, NY, USA).

### 5.7 Patient Specimens

Written informed consent was obtained from all the patients. 393 patient specimens were derived from patients underwent radical or partial nephrectomy or kidney biopsy at Xinhua Hospital between 2015 to 2025. All tumor specimens were pathologically confirmed ccRCC. After resection, the samples were immediately frozen in liquid nitrogen and stored at −80°C for further studies. Specimen collection for research purposes was approved by Clinical Research Ethics Board of Xinhua Hospital (Approval No. XHEC‐D‐2026‐059, XHEC‐C‐2021‐145‐1, XHEC‐C‐2025‐004‐1).

### 5.8 Cell Culture

786O, 769P, and 293T cells were obtained from Cell bank of typical culture preservation Committee of Chinese Academy of Sciences. 786O and 769P cells were cultured in RPMI 1640 (Gibico) while 293T cells were cultured in DMEM (Gibico), supplemented with 10% fetal bovine serum (Gibico). The PTBP1 knockout cell line was generated using the CRISPR‐Cas9 system.

### 5.9 Immunohistochemical (IHC) Assay

FFPE tissue sections were deparaffinized, rehydrated, and subjected to antigen retrieval in citrate buffer (pH 6.0) at 95°C for 20 min. Endogenous peroxidase was blocked with 3% H_2_O_2_, followed by blocking with 5% BSA for 1 h. Sections were incubated with primary antibody, then with HRP‐conjugated secondary antibody. Staining was visualized using DAB, and sections were counterstained with hematoxylin. Images were captured under a light microscope.

IHC score was evaluated by two independent pathologists blinded to the clinical data. The H‐score was calculated. Cases with an H‐score ≥ 150 were defined as “high expression” and those below as “low expression”.

### 5.10 RNA Preparation and Real‐Time Quantitative PCR

RNA from was extracted by Trizol (Invitrogen) according to the instructions. Extracted RNA was reverse transcribed using HiScript II One Step RT‐PCR Kit (Vazyme, China). RT‐PCR ChamQ SYBR qPCR Master Mix (Vazyme, China) was used for qPCR according to the instructions.

### 5.11 Western Blotting Assay

Total protein was extracted from the cultured cells or specimens using RIPA buffer according to manufacturer's protocol, and resolved by SDS‐polyacrylamide gels, and then transferred to PVDF membranes. Finally, the PVDF membranes were analyzed for chemiluminescence development. Antibodies used in research were listed in Table S1.

### 5.12 Electrophoretic Mobility Shift Assay (EMSA)

In‐vitro transcription template is synthesized by PCR and purified. RNA was synthesized by T7 High Yield RNA Transcription Kit (Vazyme, China) according to the instructions. PTBP1 recombinant protein was purchased from CUSABIO. RNA and protein were incubated in binding buffer for 30 min at room temperature. The reaction mixed were loaded onto 10% native agarose gels and electrophoresed on ice. The gels were steeped in 2×gelred buffer for 1 h to stain RNA and then scanned using an ultraviolet emitter. After that, the gels were steeped in FastBlue (Biosharp, China) to stain protein for 2 h and then photographed. Equal doses of BSA were used as negative control.

### 5.13 Proliferation and Drug Sensitivity Assay

The proliferation and EVE sensitivity of ccRCC cells under specified conditions was detected with CCK8 kit (Dojindo Kumamoto, Japan) according to the manufacturer's instructions. The media were replaced with fresh ones before testing. 10 µL CCK‐8 was added to each well, and the samples were cultured at 37°C for 2 h. OD values were measured at 450 nm absorbance using a microplate reader. Everolimus and PT109 was purchased from MCE.

### 5.14 Transwell Assay

About 1 × 10^5^ of cells were plated in the upper chambers of 24‐well transwell plates (Corning, USA) in FBS‐free medium, and complete medium with 10% FBS was deposited in the lower chambers to serve as a chemo‐attractant. For in vitro migration assay, cells that passed through the Transwell filter after 12 h were remained and stained by crystal violet. For in vitro invasion assay, transwell membranes were coated with Matrigel prior to plating cells, cells that passed through the Matrigel membrane and transwell filter after 24 h were stained and calculated. The relative number of passed cells represented the cells’ ability to migrate or invasion.

### 5.15 Animal Experiments

For subcutaneous model, 5 × 10^5^ Renca cells were injected into balb/c mice, or 5 × 10^6^ 786O cells were injected into NOD/SCID mice at a single site. Tumor sizes were measured and calculated every 3–5 days. At the study endpoint, mice were executed and tumors were excised and weighted. For the treatment group, EVE was administered by gavage for 0.25 mg/kg from day15. The animal experimental procedures were approved by the Ethics Committee of Xinhua Hospital (Approval No. XHEC‐F‐2026‐022).

### 5.16 Statistical Analysis

All data were analyzed by GraphPad Prism V.9.0 and presented as the mean ± SD. Statistical tests for data analysis included Student's *t*‐test and Spearman correlation analysis. *P* values were determined by a two‐tailed Student's *t*‐test for comparing two groups. *P* values were indicated with *p < 0.05, **p < 0.01, and ***p < 0.001 on graphs. Graphs not labeled with an asterisk indicate that the differences between the test groups and the control groups were not statistically significant. All in vitro experiments were repeated with at least three replicates.

## Author Contributions

X.W.P., H.F.Z., M.C.L., and W.Z. conceived and designed the study. X.W.P., M.C.L., and J.L.Z. performed bioinformatic analysis. H.F.Z. and M.C.L. performed experiments. H.F.Z., M.C.L., J.L.Z., and W.Z. analyzed and interpreted the data. H.B.Z., D.H.L., Y.F.T., T.Y.Y., Y.F.L., Z.C.L., W.J.M., Y.B.Z., J.G.L., and Z.X.G. assisted with data curation. H.F.Z. drafted the manuscript. X.W.P., H.F.Z., M.C.L., and K.Q.D. reviewed and finally approved the version to be published. H.Z. and K.Q.D. provided methods and technical support. X.G.C., J.Q.Y., W.Z., and X.W.P. provided administrative, material, or financial support. All authors approved the version to be published and agreed to be accountable for all aspects of the work.

## Funding

This work was sponsored by the National Natural Science Foundation of China (No. 82330094, U25A20122); National Key Research and Development Program (No. 2024YFF1501304); Shanghai Leading Talent Program Project (LJ2024013); Shanghai Rising‐Star Program (23QC1401400); Shanghai Oriental Talent Plan Youth Program (QNJY2025090); Natural Science Foundation of Shanghai (23ZR1441300); “Cross‐Fund Project” of Xinhua Hospital (JC2025G008).

## Conflicts of Interest

The authors declare no conflicts of interest.

## Supporting information




**Supporting File**: advs77782‐sup‐0001‐SuppMat.docx.

## Data Availability

Original data generated in this study are available from the corresponding authors on reasonable request.
