# Supplementary material for: PTBP1‐Mediated Alternative Splicing of DNAJB6 Promotes Everolimus Resistance in Clear Cell Renal Cell Carcinoma via EIF4B/PKIB/AKT/mTOR Positive Feedback Loop
Source: Adv Sci (Weinh). 2026 Sep 27:e77782. Online ahead of print. doi: 10.1002/advs.77782 (PMC13616274; doi:10.1002/advs.77782)
Supplement: Supplementary file 1 [file ADVS-9999-e77782-s001.docx]

**Supplemental figure 1** (A) Dot plot of marker genes expression in 8 clusters and (B) Heatmap of correlation between 8 clusters from mice scRNA-seq. (C) Heatmap of GEVA score for immune cells pathways. (D) Featureplot of marker genes expression in 8 clusters from human snRNA-seq. (E) Dot plot of marker genes expression in 6 epithelial subclusters from human snRNA-seq. (F) The group clusters of epithelial cells. (G) Heatmap of correlation between 6 epithelial subclusters from human snRNA-seq.

**Supplemental figure 2** TCGA-KIRC database analysis on correlation between PTBP1 expression and DNA replication, Chromatin organization, Chromatin remodeling, mRNA splicing and alternative mRNA splicing pathways.

.

**Supplemental figure 3** (A) Knockout or knockdown of PTBP1 inhibited the proliferation ability of ccRCC cells. (B) Knockout or knockdown of PTBP1 inhibited the migration and invasion ability of ccRCC cells. (C) Quantitative results of migration and invasion assay. Statistical tests involved: **P* < 0.05, ***P* < 0.01, ****P*<0.001, Student’s t-test; Data are expressed as mean±SD, n = 3.

**Supplemental figure 4** (A) Densitometric quantification of the Western blot bands shown in Figure 5A. (B) Overexpression of DNAJB6b rescued the AKT/mTOR signaling inhibition caused by PTBP1 depletion. Statistical tests involved: **P* < 0.05, ***P* < 0.01, ****P*<0.001, Student’s t-test; Data are expressed as mean±SD, n = 3.

**Supplemental figure 5** (A) Overexpression of DNAJB6b promoted EVE insensitivity of ccRCC cells.

**Supplemental figure 6** GST pull-down assay. Cell lysates expressing FLAG-tagged EIF4B were incubated with purified GST-tagged DNAJB6a/b or GST alone (as a control), followed by pull-down with glutathione-Sepharose beads. Bound proteins were detected by western blot with anti-FLAG and anti-GST antibodies.

**Supplemental figure 7** (A) Predicted Aligned Error (PAE) plots showing predicted positional error between DNAJB6b CTD and EIF4B RRM in models rank_1 rank_5. (B) Per-residue pLDDT scores across the five models, with the vertical line indicating the boundary between DNAJB6b CTD and EIF4B RRM.

**Supplemental figure 8** Densitometric quantification of the Western blot bands shown in Figure 7C (A), Figure 7D (B), Figure 7I (C) and Figure 7J (D). Statistical tests involved: **P* < 0.05, ***P* < 0.01, ****P*<0.001, Student’s t-test; Data are expressed as mean±SD, n = 3.

**Supplemental figure 9** (A) DNAJB6b overexpression blocked EVE sensitivity, whereas PKIB knockdown could enhance it. (B) Western blot on whole signaling pathway with patients’ specimens.

**Supplemental figure 10** Densitometric quantification of the Western blot bands shown in Figure 8D (A) and Figure 8I (B). Statistical tests involved: **P* < 0.05, ***P* < 0.01, ****P*<0.001, Student’s t-test; Data are expressed as mean±SD, n = 3.

**Supplemental table 1. Antibodies information**

| **Antibody** | **Catalog** |
| --- | --- |
| anti-PTBP1 (for CLIP) | CST, #57246 |
| anti-PTBP1 (for WB and IHC) | Abcam, ab133734 |
| anti-DNAJB6 | Abcam, ab198995 |
| anti-EIF4B | Abcam, ab68474 |
| anti-AKT | Abcam, ab235958 |
| anti-phospho-AKT | Abcam, ab222489 |
| anti-P70S6K | CST, #2708 |
| anti-phospho-P70S6K | CST, #9234 |
| anti-puromycin | Abcam, ab315887 |
| anti-PKIB | Abcam, ab233521 |
| anti-ORC1 | Abcam, ab85830 |
| anti-CDK16 | Abcam, ab181208 |
| anti-SLC2A1 | Abcam, ab115730 |
| anti-PAK4 | Abcam, ab62509 |
| anti-TGFB1 | Abcam, ab215715 |
| anti-BCL2 | Abcam, ab182858 |
| anti-BCL6 | Abcam, ab172610 |
| anti-MCL1 | Abcam, ab32087 |
| anti-cMYC | Abcam, ab32072 |
| anti-Flag | CST, #14793 |
| anti-β-Actin | Abcam, ab8227 |
